# Study of QCL Laser Sources for the Realization of Advanced Sensors

**DOI:** 10.3390/s150819140

**Published:** 2015-08-05

**Authors:** Giuseppe de Risi, Lorenzo Luigi Columbo, Massimo Brambilla

**Affiliations:** 1 Dipartimento Interareneo di Fisica, Università degli Studi e Politecnico di Bari, via Amendola 173, I-70126 Bari, Italy; E-Mails: giuseppe.derisi@gmail.com (G.R.); massimo.brambilla@uniba.it (M.B.); 2 CNR—Istituto di Fotonica e Nanotecnologie UOS Bari, via Amendola 173, I-70126 Bari, Italy

**Keywords:** quantum cascade laser, optical feedback interferometry, nano displacement sensing, laser sensors

## Abstract

We study the nonlinear dynamics of a quantum cascade laser (QCL) with a strong reinjection provided by the feedback from two external targets in a double cavity configuration. The nonlinear coupling of interferometric signals from the two targets allows us to propose a displacement sensor with nanometric resolution. The system exploits the ultra-stability of QCLs in self-mixing configuration to access the intrinsic nonlinearity of the laser, described by the Lang–Kobayashi model, and it relies on a stroboscopic-like effect in the voltage signal registered at the QCL terminals that relates the “slow” target motion to the “fast” target one.

## 1. Introduction

In the last decade, quantum cascade lasers (QCLs), which represent compact, high power, highly coherent, widely tunable laser sources in the mid-infrared to terahertz range of the electromagnetic spectrum, have been extensively used in a number of sensing applications, like imaging, medical diagnosis and spectroscopy [1].

With respect to conventional bipolar semiconductor lasers, continuous wave (CW) emission in QCLs is much more stable against strong optical feedback provided by an external target in the so-called self-mixing (SM) configuration. As we recently demonstrated, this follows from the absence of relaxation oscillations due to the ultra-fast carriers recombination and to the small value of the linewidth enhancement factor (or *α* factor) [2,3].

Then, QCLs can be exploited to realize robust, detectorless, real-time sensors when an external target provides back-reflected radiation, which induces changes in the emitter properties (field intensity, compliance voltage at laser contacts). The modified signal hence carries information about corresponding variations of the target complex reflectivity and its displacement with respect to the laser exit facet [4]. Fields of applications range from coherent imaging [5] to motion tracking [6] and material processing [7].

The search for displacement sensors based on optical interferometry with nanometric resolution has been recently triggered by the enormous development of nanoscale technology, even in systems working at longer wavelengths (from mid-infrared to terahertz). So far, most of the systems showing these performances employ “off-line” signal post-processing to overcome the half wavelength intrinsic resolution of standard “on-line” fringe counting techniques [8,9,10]. A prototype system able to measure “on-line” nanometer-size amplitude displacements has been proposed in [11], based on a modification of the standard self-mixing scheme, namely a differential optical feedback interferometry in a two-laser configuration.

**Figure 1:**
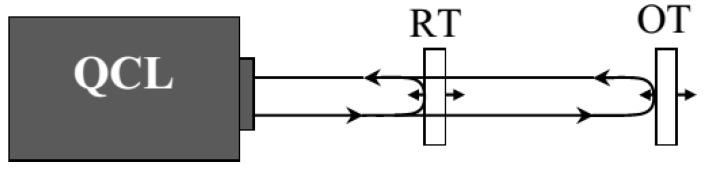
Scheme of the proposed sensor.

Here, we provide a proof-of-principle demonstration of a nanoscale position-sensing system based on the nonlinear dynamical response of a QCL subject to strong optical feedback. We refer in particular to the collinear double-arm configuration sketched in Figure 1 where optical feedback is provided by a slow object target (OT) with constant and unknown speed and a fast reference target (RT) with constant, controlled speed [12]. In the strong feedback regime, we show that the fast switching fringes in the SM signal typically associated with the RT motion are characterized by additional sub-features carrying information about the OT motion. As illustrated in detail in Section 2 and Section 3, this allows for a denser sampling of the OT fringes, which, in turn, leads to a displacement measure with a resolution much smaller than λ/2. With respect to our first results reported in [13], we present here a radically-improved version of the OT motion retrieval algorithm that allows us to reduce the estimated sensors resolution down to a few nanometers. We also provide an evaluation of the role of the feedback strength and discuss the role of the QCL fluctuations as a source of errors in the sensing procedure. We believe that the a super-resolved displacement sensor, like the one proposed here, may be of interest also in other applicative fields, such as QCL-based 3D imaging systems for medical and material processing, where the measurement of the separation between layers orthogonal to the optical axis is essential [14].

A theoretical analysis of the QCL subject to optical feedback from two independent targets is presented in Section 2. In Section 3, we describe in detail a numerical fitting procedure able to extract in real time the information about the object target displacement with a resolution of a few nanometers, which corresponds to ∼*λ*/1000. The numerical limits in the attainable resolution and the range of application of the above algorithm are discussed in Section 3.2 and Section 3.3. In Section 3.4, we estimate the influence of the finite laser linewidth on the proposed sensor accuracy. Finally, Section 4 is devoted to the conclusions and a discussion of a possible extension of our approach.

## 2. Theoretical Section: Self-Mixing in QCL with a Double External Cavity

The sensor scheme is sketched as in Figure 1, where a partially-transparent reference target moving with a known constant velocity $v_{r}$ is inserted in the external cavity formed by the QCL and the object target that translates with an unknown constant velocity $v_{o}$ < $v_{r}$.

In the single longitudinal mode, slowly varying envelope approximations and single reflection in the external cavity, the behavior of a QCL under optical feedback from two independent targets can be described by an extended version of the Lang–Kobayashi (LK) equations [4] that account for an external double cavity [15]:

(1) $$\begin{array}{ccc} \frac{dE(t)}{dt} & = & \frac{1}{2}\left(1+i\alpha \right)\left(N\left(t\right)-1\right)E\left(t\right)+\frac{k_{o}\tau_{p}}{\tau_{c}}E\left(t-\tau_{o}\right)e^{-i\omega_{0}\tau_{o}}+\frac{k_{r}\tau_{p}}{\tau_{c}}E\left(t-\tau_{r}\right)e^{-i\omega_{0}\tau_{r}} \end{array}$$

(2) $$\begin{array}{ccc} \frac{dN(t)}{dt} & = & \gamma \left(I_{p}{-N\left(t\right)(1+|E\left(t\right)|}^{2})\right) \end{array}$$

where the adimensional field *E*, carriers’ density *N* and pump intensity $I_{p}$ are scaled as in [2], and the time *t* is expressed in units of the photon lifetime $\tau_{p}$. Moreover, *α* is the linewidth enhancement factor; $\tau_{c}$ is the laser cavity round trip time; $\omega_{0}$ is the solitary laser frequency (equal to the laser cavity resonance taken as the reference frequency); *γ* is the photon-to-carrier lifetime ratio. The feedback strength parameters $k_{i}$ with i=o,r depend on the effective fraction of the field back-reflected from OT and RT that re-enters the laser cavity and the reflectivities of the external targets and of the laser exit facet. The delays $\tau_{i}$ change in time due to the target motions, so that $\tau_{i}=2L_{i}/c=2\left(L_{0,i}+v_{i}t\right)/c$ where $L_{0,i}$ represents the target initial position.

Looking for CW solutions of Equations (1) and (2), we get the following expressions for the CW QCL frequency $\omega_{F}$ and the associated difference ΔN between the carriers’ density in the presence of feedback and its value in the free running laser case [12]:

(3) $$\begin{array}{ccc} \omega_{F} & = & \omega_{0}-\frac{k_{o}\tau_{p}}{\tau_{c}}\sqrt{1+\alpha^{2}}\sin\left(A_{o}+\omega_{o}t+\arctan\alpha \right)-\frac{k_{r}\tau_{p}}{\tau_{c}}\sqrt{1+\alpha^{2}}\sin\left(A_{r}+\omega_{r}t+\arctan\alpha \right) \end{array}$$

(4) $$\begin{array}{ccc} \Delta N & = & -2\frac{k_{o}\tau_{p}}{\tau_{c}}\cos\left(A_{o}+\omega_{o}t\right)-2\frac{k_{r}\tau_{p}}{\tau_{c}}\cos\left(A_{r}+\omega_{r}t\right) \end{array}$$

where $A_{i}=2L_{0,i}\omega_{F}/c$ and $\omega_{i}=2v_{i}\omega_{F}/c$. Hence, the temporal evolution of the quantity ΔN=ΔN(t), which is proportional to the voltage offset at the QCL terminals and, thus, represents the experimentally-accessible SM signal [13], contains information about speed (and consequently, position) of both targets. From this, it follows that an explicit solution of Equations (3) and (4) would allow one, in principle, to determine the value of the OT velocity and, thus, the OT displacement. Due to the highly implicit character of the transcendent Equation (3), this is analytically impossible, and numerical methods have to be used to recover the information about the OT from the ΔN time trace.

**Figure 2:**
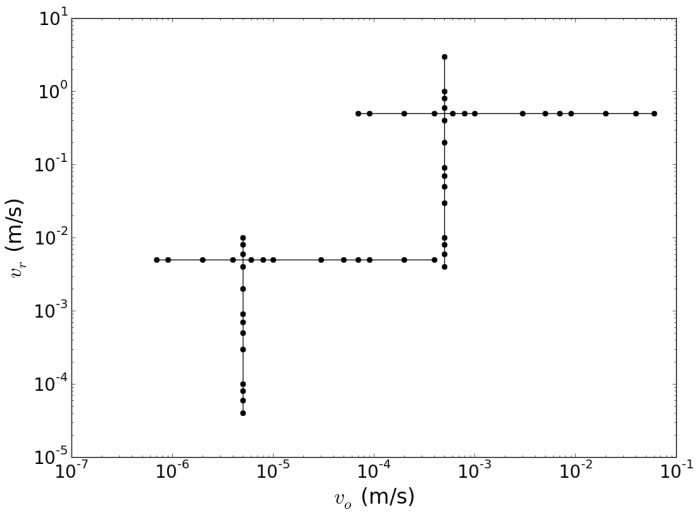
Two-dimensional parameter space of the simulations. Dots indicate the values of $v_{r}$ and $v_{o}$ used in the various simulations.

For this purpose, we simulate the evolution of the SM function ΔN, by numerically solving Equations (3) and (4) for the values of $v_{r}$ and $v_{o}$ indicated by dots in the parameter space depicted in Figure 2. The other parameters used in our simulations are typical for a mid-infrared QCL and are reported in Table 1.

**Table 1:** Physical parameters used in the simulations of the Lang–Kobayashi (LK) equations.

$\tau_{p}$	$\tau_{c}$	*α*	$\omega_{0}$	$L_{0,r}$	$L_{0,o}$
100 ps	35.6 ps	2	302 THz	$1.5\times 10^{-3}$ m	$2.5\times 10^{-3}$ m

### 2.1. Nonlinear Frequency Mixing

**Figure 3:**
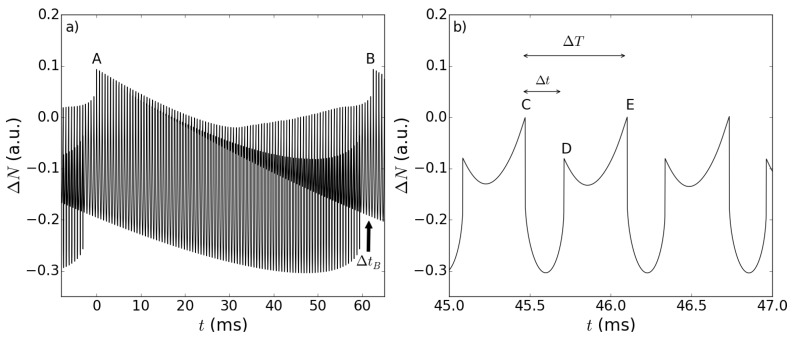
Typical temporal trace of the carrier density difference ΔN as a function of time. Reference and object target velocities are $v_{r}=5.0\times 10^{-3}$ m/s and $v_{o}=5.0\times 10^{-5}$ m/s, respectively, while feedback parameters are $k_{r}=0.029$ and $k_{o}=0.025$. (**a**) A complete slow fringe, delimited by Points A (at t=0 ms) and B (at t=62 ms), is shown, so that the faster modulation induced by the reference target is evident; in addition, the time interval of sub-feature disappearance ($\Delta t_{B}$), introduced in Section 3.3, is represented. (**b**) A close up, for a shorter time interval, of the temporal trace shown in (a), where the fast fringe can be identified (between Points C and E), with its associated time interval, denoted by ΔT. The sub-feature is delimited by Points D and E, and the time interval Δt between two consecutive sub-features is the physical quantity on which the sensing scheme is based. The other parameters are reported in Table 1.

**Figure 4:**
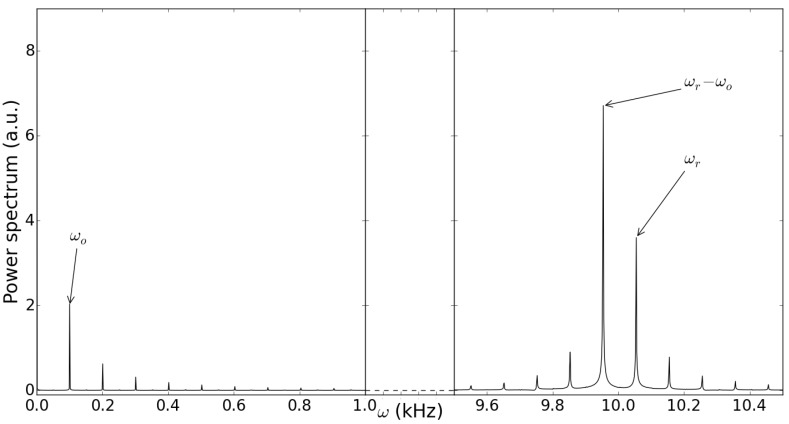
Fourier transform of ΔN(t) represented in Figure 3a.

An example of the carrier density difference ΔN(t) obtained from our simulations is represented in Figure 3. Its temporal trace exhibits two distinct modulations, which show the peculiar interference fringes of the lasers operating in SM configuration: fast fringes (from Mark *A*to Mark *B* in Figure 3a) on the scale of a few $10^{-1}$ ms, modulated by slower fringes (see also Figure 2a in [12]). While one could expect that they correspond to the fast and slow motions of the RT and OT, respectively, the inspection of the Fourier transform of ΔN(t), shown in Figure 4, reveals the nonlinear nature of the SM signal. In fact, the first peak, at frequency $\omega_{o}=4\pi v_{o}/\lambda_{0}=100$ Hz, is associated with the slow periodicity due to the movement of the OT; another peak at $\omega_{r}=4\pi v_{r}/\lambda_{0}=10$ kHz, is associated with the motion of the RT, but the dominant peak in the spectrum occurs at $\omega_{r}-\omega_{o}$. This shows that the feedback fields provided by the targets cause a nonlinear response in the laser, due to the intrinsic nonlinearity of the LK Equations (3) and (4). The presence of super harmonics of the mixed frequencies are another proof of this phenomenon and will be briefly discussed in Section 3.3. In the time domain, this amounts to saying that there should exist temporal features of the time trace, associated with a fast time scale (being $2\pi /\left(\omega_{r}-\omega_{o}\right)\approx 2\pi /\omega_{r}$), which carry information about the slow time scale. Since the former is associated with the RT motion and the latter to the OT motion, the introduction of the RT should allow one to gather information about the OT motion by sampling the system at a fast rate. This overcomes the limit λ/2 for the appreciable displacement when only one target is considered.

More interestingly, again, if the values of the feedback parameters are high enough [13], novel sub-features can be seen in the time trace (see Figure 3); such sub-features are the fingerprint of the two-target scheme, and their temporal characteristics are linked to the frequency combination arising from the nonlinear response described above. The study of these sub-features will be the main focus of the next section, so it will be worth sketching an interpretation about their origin and evolution. For moderate and strong feedback levels, multiple solutions of Equation (3) may exist, resulting in different possible continuous wave solutions (or modes), and following the well-established literature [4], we assume that the actual lasing mode is the “maximum gain mode” (MGM), *i.e.*, the one that minimizes ΔN [4] (simulations of the dynamical LK equations and comparison with experiments [13] show that this assumption is correct in the operating conditions that we considered). During the target evolution, it is possible that a new mode with higher gain becomes available, thus causing the lasing frequency to switch on this new mode. In the case of feedback from a single target, this leads to the typical sawtooth behavior of the ΔN temporal trace: its discontinuity represents the switching of the lasing frequency from one mode to another. In the double target setup we are considering, because of the frequency mixing described by Equation (3), the appearance of a new MGM is influenced by both target feedbacks. In particular, during the movement of the RT on a timescale comparable to the fast fringe period, there can be a time lapse (typically shorter than the fast fringe period itself) in which the new MGM exists. During this time, the laser frequency switches between the two different modes, inducing a sudden transition in the frequency and carrier density, leaving a peculiar and easily recognizable fingerprint in the time trace, which we call the “sub-feature”. The duration of these sub-features, and, conversely, the time interval between two subsequent sub-features, also depends on the OT motion, as indicated by the fact that it varies from one fast fringe to another, within the periodicity of the slow fringe. This is precisely the temporal feature on the basis of which the slow motion of OT can be tracked in the fast fringes, provided that one is able to extract the dependence on the OT velocity encoded in the duration of the sub-features. Referring to Figure 3, the slow fringe is the part of the temporal trace between Points *A* and *B* in Figure 3a, while the fast fringe is the part between Points *C* and *E* in Figure 3b (its duration is indicated by ΔT), and the sub-feature is the part between Points *D* (the left cusp) and *E* (the right cusp); finally, with Δt, we denote the time interval between the edges of two consecutive sub-features, namely between Points *C* and *D* in Figure 3b.

## 3. Super-Resolved Displacement Sensor

In this section, we first describe the procedure for analyzing ΔN(t) and for identifying the relevant temporal marks of the sub-features described above. Then, we present the numerical scheme used to relate such data to the OT displacement.

As we have discussed in the previous section, information about the movement of RT and OT is linked together in the features of the temporal trace via Equation (4). We performed an extensive fitting procedure, to relate the position of the OT to the time interval Δt between the cusps of two subsequent sub-features. The foundations of this method have been presented in [13]. Here, we radically improve it and correlate it with an analysis of the achievable precision in relation to the calibration stage. We will make use of the increased number and accuracy of the simulations and of the broader parameter space(see Figure 2).

### 3.1. Numerical Approach

All of the simulations show that the sub-features emerge at the beginning of the slow fringe. Their duration $t_{E}-t_{D}=\Delta T-\Delta t$, when compared to the fast fringe duration ΔT, is very small at the beginning, and it grows as the OT moves, until, near the end of the slow fringe, it becomes of the same magnitude as ΔT. This actually results in the vanishing of the sub-features for a short period of time (indicated by $\Delta t_{B}$ in Figure 3a) that terminates at the slow fringe jump, after which they reappear in the next slow fringe; of course, the dynamical behavior of Δt across the slow fringe is complementary). We now illustrate how it is possible to relate the change in the time lapse Δt to the position of the OT. In our simulation, the OT velocity $v_{o}$ is fixed, so we can define a “theoretical” position of the OT as given by the simple formula:

(5) $$S_{th}=v_{o}t$$

The time trace is thus sampled to extract the values of Δt at different instants of time; at each time, we assume that the position of the OT is given by $S_{th}$, and we will perform a best fit analysis to find a function that relates the position $S_{th}$ to Δt. Since, as we mentioned above, there can be a short period across the slow fringe change in which the sub-features disappear, the fitting procedure can be performed in principle only along a single slow fringe. In the next subsection, we will tackle the problem of overcoming this limit, because an actual sensor must be capable of operating on an (ideally) arbitrarily long period, while at this stage, we will limit our analysis to a single slow fringe.

We have developed an algorithm capable of identifying and classifying (potentially in real time) all of the critical points of the temporal trace labeled in Figure 3, so that we can register the time lapses between them. We set the origin of time at the first left cusp identified by the analysis algorithm, and we take the reference times $t_{n}$ at each subsequent left cusp, while, as already mentioned before, we register the time lapse $\Delta t_{n}$ that occurs between the right cusp of the (n-1)-th sub-feature and the left cusp of the *n*-th one. Upon varying the fit on a broad basis of polynomial and transcendent functions, we found that a quadratic dependence works surprisingly well in approximating such dependence, and the candidate test function can be cast as:

(6) $$S_{fit}\left(\Delta t_{n}\right)=C_{2}\Delta t_{n}^{2}+C_{1}\Delta t_{n}+C_{0}$$

The least squares method can be applied to evaluate the coefficients $C_{i}$ for all of the simulations. Of course, coefficients $C_{i}$ change for simulations at different $v_{r}$, because a larger $v_{r}$ implies a reduced ΔT and, thus, Δt. A proper interferometric sensor cannot suffer from such “reference arm” dependence, so the next step is to make the $v_{r}$ dependence explicit in Equation (6). For such a purpose, a new best fit procedure was carried out, resorting to the sets of simulations with varying $v_{r}$. The coefficients $C_{2}$ and $C_{1}$ have been found to have a quadratic and a linear dependence on $v_{r}$, respectively, while $C_{0}$ is independent of $v_{r}$. The general formula we sought, which is supposed to hold for a wide range of the $\left(v_{r},v_{o}\right)$ plane, could then be cast as:

(7) $$S_{phen}\left(\Delta t_{n}\right)=-\gamma_{2}\frac{v_{r}^{2}}{\lambda_{0}}\Delta t_{n}^{2}-\gamma_{1}v_{r}\Delta t_{n}+\gamma_{0}\frac{\lambda_{0}}{2}$$

In this formula, we insert the solitary laser wavelength $\lambda_{0}=2\pi c/\omega_{0}$ to make the dimensions of the coefficients clear. To determine the parameters $\gamma_{i}$ from the entire set of simulations, we have evaluated the parameters $C_{i}$ for all of the complete slow fringes in each simulation, and we have extracted the values of the $\gamma_{i}$, along with their errors, by using their expressions as functions of the $C_{i}$. Each value obtained in this way has been treated as an independent detection of the “true” $\gamma_{i}$ value, so that its best estimate is the weighted means evaluated from all of the occurrences. We found:

(8) $$\begin{array}{ccc} \gamma_{2} & = & 1.0197\pm 0.0017\\ \gamma_{1} & = & 0.6696\pm 0.0009\\ \gamma_{0} & = & 0.9375\pm 0.0002 \end{array}$$

and these parameters are constant within the assumptions described so far.

### 3.2. The Sensing Procedure: Methods, Sensitivity and Limits

Having obtained the relation defined in Equation (7), it can be used to determine the displacement of the OT for any velocity pair $\left(v_{r},v_{o}\right)$ compatible with the limits of validity, which are going to be discussed here. First of all, the model was built on the stationary solutions of the LK equations, so we must ensure that the evolution of the target is adiabatic, *i.e.*, the laser system has enough time to reach a stationary state as the targets move. The slowest time on which the system evolves is of the order of tens of nanoseconds [2,3], while the evolution timescale of the carrier density difference associated with the motion of the RT is of the order of the fast fringe period, $\Delta T≃\lambda_{0}/2v_{r}$. Therefore, adiabaticity requires that $v_{r}<<v_{r,max}$ with $v_{r,max}≃100$ m/s. In our simulations, the maximum value for the RT velocity was thus set to 5 m/s.

The reliability of the phenomenological relation Equation (6) can be assessed by appreciation of the normalized root mean square deviation for each fitting procedure:

(9) $$\mathrm{RMS}=\frac{1}{𝒩-3}\sum_{n}{\left(\frac{S_{th}\left(\Delta t_{n}\right)-S_{fit}\left(\Delta t_{n}\right)}{S_{th}\left(\Delta t_{n}\right)}\right)}^{2}$$

where *𝒩* is the number of sub-features identified by the algorithm. We observed that its value was very small (of the order of a few $10^{-4}$) as long as the ratio $v_{o}/v_{r}$ was well above $10^{-4}$. Near this threshold, it jumped abruptly to values of the order of $10^{-1}$, thus indicating that the assumption of a quadratic dependence is no longer valid. In order to be conservative, in the evaluation of the parameters $\gamma_{i}$ in Equation (8), we have used only simulations with $v_{o}/v_{r}\geq 0.5\times 10^{-3}$. The smallest displacement that can be observed depends on the time between two subsequent observations of Δt, which is of the order of the fast fringe periodicity, $\Delta T≃\lambda_{0}/2v_{r}$, so that, in principle,

(10) $$\Delta S≃\frac{v_{o}}{v_{r}}\lambda_{0}$$

**Figure 5:**
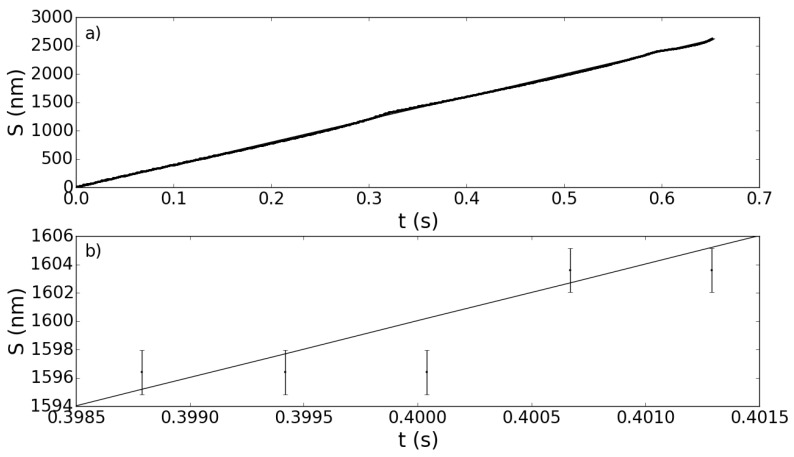
Plot of the position of the object target (OT) target S(t) *versus* time, for a simulation with $v_{r}=5\times 10^{-3}$ m/s and $v_{o}=4\times 10^{-6}$ m/s. The other parameters are as in Figure 3. The solid line represents the theoretical position $S_{th}$ (see Equation (5)). The dots mark the position $S_{phen}$ as predicted by the phenomenological Equation (7) and the associated errors $\sigma_{S}$ as given by Equation (11). (**a**) The representation over the entire period of a slow fringe; (**b**) a close-up in which the accuracy of $S_{phen}$ in retrieving $S_{th}$can be appreciated.

Since, as we discussed above, the sensing procedure allows $v_{o}/v_{r}≃10^{-3}$, the scheme we are proposing should be capable of reaching a sensitivity of $\lambda_{0}/1000$, which is of order of a few nanometers. An example of the results that can be obtained with the sensing procedure described so far is given in Figure 5.

**Figure 6:**
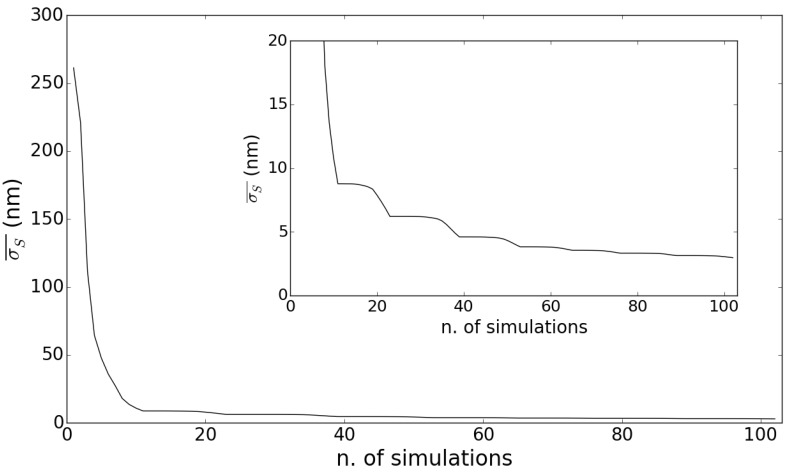
Plot of the mean error on the determination of the OT position ($\bar{\sigma_{S}}$) as a function of the number of simulations used to determine the $\gamma_{i}$ coefficients. The inset focuses on the relevant range of values for $\bar{\sigma_{S}}$ and shows that it actually reaches the nanometer range.

It is evident by inspection of the close-up, Figure 5b, that the potential measurements of the OT are well within the 10-nm deviation from the theoretically-expected values (the solid line). Of course, this result depends crucially on the accuracy in determining the coefficients $\gamma_{i}$ of Equation (8). In fact, we can assume that the error on the determination on the OT displacement due to uncertainties in the $\gamma_{i}$ parameters is given by applying the error propagation law to the phenomenological Equation (7):

(11) $$\sigma_{S}=\sqrt{\sum_{i}{\left(\frac{\partial S}{\partial \gamma_{i}}\right)}^{2}\sigma_{\gamma_{i}}^{2}}=\sqrt{{\left(\frac{v_{r}^{2}}{\lambda_{0}}\Delta t_{n}^{2}\right)}^{2}\sigma_{\gamma_{2}}+{\left(v_{r}\Delta t_{n}\right)}^{2}\sigma_{\gamma_{1}}+{\left(\frac{\lambda_{0}}{2}\right)}^{2}\sigma_{\gamma_{0}}}$$

and this error must be smaller than the expected sensitivity given by Equation (10). Increasing the number of simulated slow fringes, on which the determination of the $\gamma_{i}$ parameters is performed, will result in better accuracy. To check this, we have evaluated the mean error $\bar{\sigma_{S}}$ as a function of the number of simulations. The result is shown in Figure 6.

As we can see, the curve falls quite rapidly below 10 nm and then tends to saturate around 5 nm. This confirms that the $\gamma_{i}$ parameters can be evaluated with sufficient precision for our sensing scheme to reach nanometric sensitivity. The values reported in Equation (8) have been obtained using a set of 100 simulated slow fringes.

In a realistic sensor, the determination of the $\gamma_{i}$ parameters and their uncertainties is based on a calibration procedure where targets with known velocities are employed. The calibration should be performed for a set of pairs $\left(v_{r},v_{o}\right)$ conveniently distributed in the parameter space according to the guidelines discussed in the first part of this subsection. This calibration needs to be done only once, since the parameters determined in this way should remain valid for a range of at least three orders of magnitude in both target velocities. The number of calibration runs determines the accuracy of the value obtained for the $\gamma_{i}$ parameters and, consequently, the sensitivity of the device. A curve representing the error on the determination of the position of the target as a function of the numbers of calibration runs should have the behavior reported in Figure 6, but other factors related to the experimental conditions may hinder the capability of achieving the predicted nanometric sensitivity. Just as an example, mechanical vibrations of the set-up, irregularities in the motion provided by the step motor driving the RT and/or OT and current fluctuations in the power supply of the laser are all obvious sources of errors in the measure. While such sources can be tamed in principle, we will consider the effect of a more intrinsic limitation in Section 3.4.

Another issue to be addressed is how to circumvent the detection limitation to a single slow fringe due to the disappearance of the sub-feature discussed above. Our simulations proved that the number of fast fringes not exhibiting a sub-feature occurs invariably at the end of a slow fringe, and it decreases with increasing feedback strengths $k_{o}$ and $k_{r}$. In any case, in the time lapse $\Delta t_{B}$, our algorithm is “blind” to the target motion. A sufficiently high feedback should limit this interval to a small fraction of the slow fringe period, and during this period, the OT displacement can be extrapolated via the simple formula $\Delta S_{blind}=v_{l}\Delta t_{B}$, where $v_{l}$ is the last detected OT velocity, just before sub-feature disappearance. This simple method has been experimentally tested in [13] and proven to be sufficiently accurate within experimental uncertainties. In the next subsection, we will discuss in detail this and other aspects of the sensing procedure related to the feedback level.

Finally, let us stress that, in principle, the sensitivity could be pushed further beyond, since the limiting ratio $v_{o}/v_{r}≃0.5\times 10^{-3}$ is set by the failure of the quadratic fit proposed in Equation (7). For lower values, new sets of simulations and a more refined fitting procedure might yield a reliable relation $S_{phen}\left(\Delta t_{n}\right)$. In any case, the necessity to detect a sufficient number of slow fringes and of densely sampling each fast fringe might increase the requirement in terms of bandwidth and the buffer memory of the electronics reading out the voltage at the QCL contacts.

### 3.3. The Role of the Feedback Parameters in the Sensing Scheme

As was shown in Section 2.1, the key element for the sensor proposed in this work is the high feedback level, which ensures, in the spectral domain, the nonlinear frequency coupling and, in the time domain, the appearance of the sub-features, whence our algorithm extracts the information of the OT motion with nanometric accuracy. We stress that this feature is critical of the nonlinear dynamics of the laser in providing the coupling and of the QCL in particular, since this emitter can sustain large feedback without entering chaotic regimes [2]. We will now illustrate in some detail the role of the feedback strength on the system dynamics.

**Figure 7:**
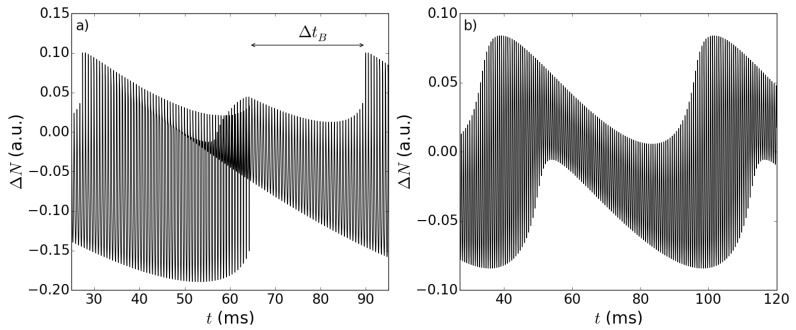
Plot of ΔN(t) for two different levels of feedback with fixed ratio $k_{r}/k_{o}$. The other parameters are as in Figure 3. (**a**) The feedback values are $k_{r}=0.0180$ and $k_{o}=0.01575$. Notice that the sub-features are present only in (approximatively) the second half of the slow fringe, as indicated by the time lapse $t_{B}$. The temporal trace (**b**), obtained using $k_{r}=0.0080$ and $k_{o}=0.007$, has no sub-features at all.

We analyzed the system behavior when the feedback levels $k_{r}$ and $k_{o}$ are changed, while keeping the ratio $k_{r}/k_{o}$ fixed. The values of the target velocities were set to $v_{r}=5\times 10^{-2}$ m/s and $v_{o}=5\times 10^{-4}$ m/s. Decreasing the feedback results in a reduction of the time fraction of the slow fringe in which the sub-features are present, as it is possible to see in Figure 7a. At even lower feedback levels, the sub-features disappear completely, as shown in Figure 7b.

**Figure 8:**
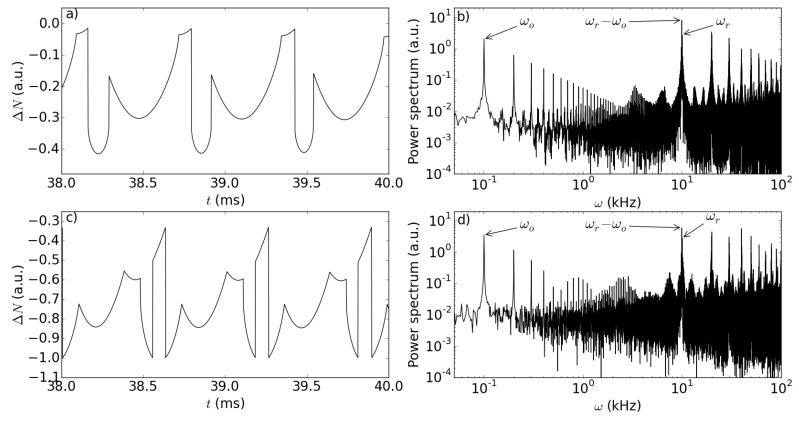
Plot of ΔN as a function of *t* (**a**,**c**) along with their corresponding Fourier transforms plotted in a log-log plane (**b**,**d**); upper part: $k_{r}=0.0404$; lower part: $k_{r}=0.1010$. The ratio $k_{r}/k_{o}$ remains fixed. The other parameters are as in Figure 3.

On the contrary, as the feedback parameters increase, new temporal features appear in the fast fringes, in the form of additional pairs of cusps with even shorter duration (see Figure 8a). While such “sub-sub-features” may be the subject of further investigations to enhance even further the potential sensitivity of our scheme, another interesting insight for this phenomenon can be gathered from inspecting the Fourier transform of ΔN(t) for different values of the feedback parameters.

Figure Figure 8b shows the Fourier transform of the temporal trace corresponding to Figure 8a. One observes a strong peak at the lowest frequency $\omega_{o}$ and its higher harmonics, while at higher frequencies, as in the case of Figure 4, the main peak occurs at the frequency difference $\omega_{r}-\omega_{o}$. Remarkably, there are several higher harmonics of this dominant note, whose peaks decrease in intensity with a power law, indicating once again the strong nonlinear character of the interaction, brought about by the laser dynamics. Interestingly, the time trace is still regularly periodic in this case, although the peak pattern is complicated by the appearance of the new cusps. Upon further increase of the feedback strength, we observe (see Figure 8d) that the background increases considerably, while the peaks at the dominant frequencies become more intense and do not decrease linearly any more; accordingly, the time trace (see Figure 8c) still exhibits regular, though complex features on the short time scale, while at longer timescales, comparable with the slow fringe period, it appears irregular, and we cannot expect to recover a relation formally similar to Equation (7).

Summing up, as concerns the proposed sensing scheme, in the general dynamical scenario of the retro-injected QCL, the most suitable feedback level one should try to set as the operational point for the sensor is the one close to the threshold of the appearance of the sub-sub-features. In this condition, in fact, the time lapse $\Delta t_{B}$ in which the sub-features disappear is the shortest possible, thus reducing the error implied by the extrapolation procedure described above.

### 3.4. Influence of the QCL Linewidth on the Sensitivity

As we mentioned in Section 3.2, several other sources of error may worsen the accuracy of Equation (7), as given by the errors on its coefficients in Equation (8), which are solely determined by the sample set on which the calibration is performed. We consider now the effect of the finite linewidth of the QCL emission. The latter is associated with phase variation induced by spontaneous emission, carrier-induced refractive index change and injection current fluctuations [16,17]. Moreover, it is known that the presence of optical feedback leads to linewidth broadening or narrowing depending on the external cavity phase shift [18]. A rigorous theoretical approach that describes all of these phenomena would consist of adding Langevin noise sources in the LK Equations (1) and (2), as is described in [17]. Here, in order to provide a simple estimation of the role of the QCL linewidth in limiting the proposed sensor accuracy, we suppose that the stochastic fluctuations of the free running laser frequency, denoted now as $\omega_{QCL}$, which follows a normal distribution centered in $\omega_{0}$ with amplitude $\Delta \omega_{QCL}$, affect the frequency $\omega_{F}$ of the reinjected QCL trough Equation (3) and, in turn, the values of ΔN as given by Equation (4). We can thus expect the fringe jumps and the sub-feature duration to fluctuate accordingly; this will induce additional uncertainties in the determination of the target displacement as evaluated in Equation (7). In an ergodic hypothesis, we consider the ensemble average of a large number of simulations with fixed $\omega_{QCL}$ as representative of the time average in the temporal evolution of fluctuating variables, as provided by the integration of Equations (1) and (2) with the inclusion of Langevin noise sources [17].

**Figure 9:**
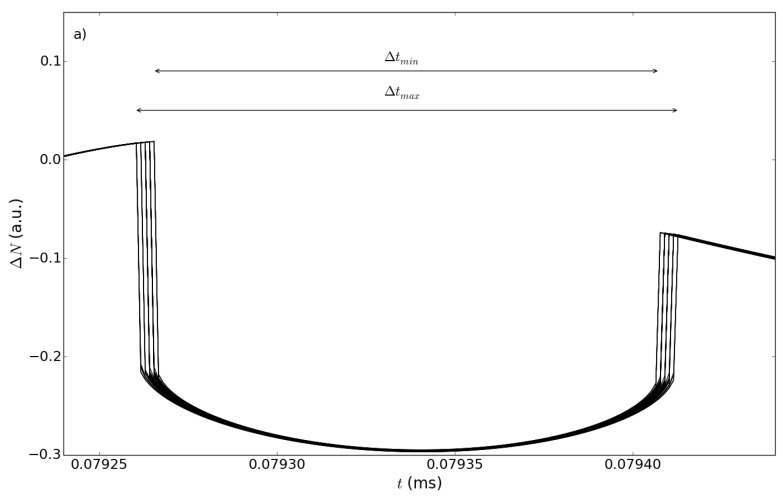
Broadening of the temporal trace ΔN(t) for a linewidth $\Delta \omega_{QCL}=10$ MHz. The plot focuses on the time lapse between two consequent sub-features, showing the maximum ($\Delta t_{max}$) and the minimum ($\Delta t_{min}$) values of Δt considered in the evaluation of the uncertainty $\sigma_{S,lw}$. The other parameters are as in Figure 3.

In particular, for the study case $v_{r}=5\times 10^{-3}$ m/s and $v_{o}=5\times 10^{-5}$ m/s and for values of $\Delta \omega_{QCL}$ ranging from 100 kHz to 10 MHz, which are in agreement with estimations reported in recent literature [19,20,21], a set of 50 determinations of $\omega_{QCL}$ was randomly generated and used to obtain 50 ΔN(t) traces, via Equation (3) (where, of course, $\omega_{0}$ was replaced by $\omega_{QCL}$). The immediate visual effect of the $\omega_{QCL}$ fluctuations introduced in this way is the jittering of the cusps delimiting the sub-features (see Figure 9). This amounts to saying that one can detect a set of $t_{C,i},i=1,...50$, corresponding to left cusps initiating a sub-feature, and another set $t_{D,j},j=1,...50$, corresponding to right cusps ending a sub-feature (see Figure 3b), which define a 50×50 array of detectable sub-feature time lapses $\Delta t_{i,j}=t_{D,j}-t_{C,i}$. Indicated as $\Delta t_{max}$ and $\Delta t_{min}$, the maximum and minimum values of $\Delta t_{i,j}$, respectively (see Figure 9), the dispersion $\sigma_{\Delta t}=\left(\Delta t_{max}-\Delta t_{min}\right)$ is considered as the error on the determination of Δt induced by the finite linewidth.

The uncertainty on the OT position has been derived with the error propagation law:

(12) $$\sigma_{S,lw}=\left(2\gamma_{2}\frac{v_{r}^{2}}{\lambda_{0}^{2}}\Delta t_{n}+v_{r}\right)\sigma_{\Delta t_{n}}$$

**Figure 10:**
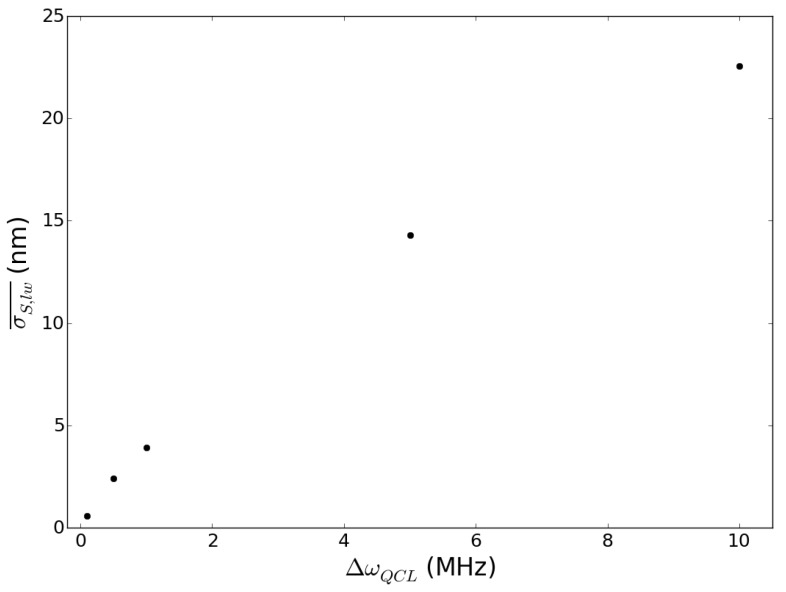
Plot of the uncertainty $\bar{\sigma_{S,lw}}$ against the quantum cascade laser (QCL) linewidth associated here with quantity $\Delta \omega_{QCL}$.

In Figure 10, we represent the behavior of the mean error $\bar{\sigma_{S,lw}}$ (obtained by averaging $\sigma_{S,lw}$ over the set of sub-features considered) as a function of the QCL linewidth. As we can see, the errors grow with linewidth according to a power law, and for $\Delta \omega_{QCL}\leq 5$ MHz, they are below 10 nm, thus comparable to those intrinsic to our deterministic method.

## 4. Conclusions and Perspectives

In conclusion, we have studied the dynamics of a QCL with retro-injection provided by two translating collinear targets, showing that the nonlinear behavior of the emitter provides a nontrivial coupling of the two feedback fields, which allows one to extract information on the slower target on a time basis linked to the interferometric fringes associated with the fast target translation. Thus, we have proposed a scheme for a real-time, nanometric displacement sensor with a resolution on the order of λ/1000, a calibration procedure for its use in a wide range of OT velocities, and we have provided an estimation of the accuracy in dependence of several factors, among which is the intrinsic linewidth of the emitter.

While this scheme could be exported in principle to any range of wavelengths, it must be noted that high levels of feedback strength are necessary to ensure a significant nonlinear response and, thus, the occurrence of the sub-features upon whose durations we based the sensor scheme. Conventional diode lasers appear to be unsuited for such scheme, since the undamped amplification of the relaxation oscillations frequency causes the emitter to enter a chaotic regime at high feedback levels [4]. On the other hand, the QCL is quite appealing, not only for the wavelength range, at which several materials of interest are transparent, but also because of the narrow linewidth, which is beneficial for the sensitivity of the present sensor scheme. We believe that our work shows how, quite generally, the introduction of an additional interferometric element (here, the moving RT) provides a second, controlled and fast “clock tick”, which allows fast sampling of an independent process (the OT translation), while the nonlinear dynamics of the laser provides the coupling between the two. It will be of course interesting to extend the present scheme to analyze arbitrary OT motions and to model more convenient RT dynamics, such as vibrations of the RT etalon, as they may be provided by a piezo controller. Other than faster RT displacements and, thus, improved basic resolution (see Equation (10)), such an extension may provide a simpler device with a reduced footprint. In this respect, entirely new knowledge must be acquired concerning the relation among RT and OT spectral features appearing in the QCL output. Work is in progress in this direction. Finally, and on a more fundamental note, it has been shown that a QCL with feedback can exhibit a multimode regime of regular oscillations, corresponding to coherent locking of modes of the external cavity provided by a reflector [3]. The inclusion of two reflectors, thus the existence of two sets of independently-tunable, external modes, might provide an “engineered” modal competition (e.g., when the two sets have free spectral range ratios in the rational or in the irrational domain) and reveal novel features in the coherent QCL dynamics.

The authors also wish to thank Francesco Paolo Mezzapesa for useful discussions.
